# Baicalin ameliorates chronic mild stress-induced depression-like behaviors in mice and attenuates inflammatory cytokines and oxidative stress

**DOI:** 10.1590/1414-431X20198434

**Published:** 2019-06-19

**Authors:** Juying Zhong, Gonghua Li, Hong Xu, Yan Wang, Mingming Shi

**Affiliations:** 1 Department of Pharmacy, Tongde Hospital of Zhejiang Province, Hangzhou, Zhejiang, China; 2 Department of Pharmacy, Elderly Care Hospital of Zhejiang Province, Hangzhou, Zhejiang, China

**Keywords:** Baicalin, Depression, Neuroinflammatory, Oxidative stress

## Abstract

The natural flavonoid glycoside baicalin (BA) produces a variety of pharmaceutical effects, particularly for psychiatric/neurological disorders. This study evaluated the behavioral and neuroprotective effects of BA in mice subjected to chronic unpredictable mild stress, a model of depression. BA (25 and 50 mg/kg) significantly increased sucrose consumption and reduced immobility times in the tail suspension and forced swim tests, demonstrating that BA alleviated depression-like behaviors. Moreover, BA reduced the levels of inflammatory cytokines, such as interleukin 1β, interleukin 6, and tumor necrosis factor α, in serum and in the hippocampus. BA also abrogated increases in NMDAR/NR2B and Ca^2+^/calmodulin-dependent protein kinase II, and the decrease in phosphorylated ERK and reactive oxygen species production in mice subjected to chronic unpredictable mild stress. These findings suggested that the antidepressive effects of BA are due to the regulation of an NMDAR/NR2B-ERK1/2-related pathway and inhibition of inflammatory cytokines and oxidative stress. Thus, BA represents a potential candidate drug for patients suffering from depression.

## Introduction

Depression is a common mental disorder in which individuals experience bodily disturbances (for example, out-of-tune embodiment and loss of bodily resonance), loss of interest, and feelings of guilt, often leading to suicidal tendencies (1,2). According to a 2012 report from the World Health Organization, 350 million people worldwide suffer from a depressive disorder, which is predicted to become the second most serious contributor to the global disease burden (after heart disease) by 2020 (3). Emerging evidence suggests that the pathological process of depression involves oxido-nitrosative stress and the neuroinflammatory cascade (4), as well as deficiencies in cell proliferation, aberrant cytokine production, and disordered neuroplasticity (5). To better understand the pathophysiology of depressive diseases, studies have employed various animal models, including the exposure of rodents to chronic unpredictable mild stress (CUMS) (6). CUMS validly and reliably induces depression-like behaviors and physiological effects that mimic neuropsychiatric disorders (7,8).

In the brain, cytokines, such as interleukin (IL)-1β, IL-6, and tumor necrosis factor alpha (TNF-α), affect neurotransmission, neuroendocrine function, and behavior (9). Inflammatory cytokines activate excitotoxic cellular cascades, resulting in apoptosis in the hippocampus and cortex, which is associated with depression-like behaviors (10). The levels of various proinflammatory cytokines are increased in patients suffering from depression, and anti-inflammatory therapy produces antidepressant effects in patients with autoimmune and inflammatory disorders (11,12). These data have given rise to the hypothesis that the pathogenesis of major depression involves inflammatory processes and brain-systemic immune interactions.

Accumulating evidence indicates that the pathophysiology of depression also involves the *N*-methyl-d-aspartate receptor (NMDAR), particularly subtype 2B (NR2B) (13). The NMDAR is an ionotropic glutamate receptor important for neuronal plasticity and memory (14), but overactivation (often associated with elevated NR2B levels) can induce neuronal damage (15). Recent studies have shown that inhibiting the overexpression of NR2B ameliorates cognitive dysfunction in rats with CUMS-induced depression (16). NR2B-containing channels predominantly signal via extracellular signal-regulated kinase (ERK) (17), a pivotal transcription regulator of the mitogen-activated protein kinase family (18). One potential effector of ERK signaling involved in CUMS-induced depression is cAMP response element binding protein, a transcription factor essential for neurogenesis, neuronal survival, and neuroplasticity (19) and, for which, the activity (20) and phosphorylation (21) are linked to the responsiveness to antidepressants.

Recent studies revealed that baicalin (BA), a type of flavonoid with no obvious adverse effects that is commonly used in traditional Chinese medicine, protects neurons from cell death and enhances neurological function following cerebral ischemia. For example, Wang et al. (22) found that BA attenuated learning and memory impairments induced by global cerebral ischemia/reperfusion. Additionally, BA eliminates depression-like behaviors induced by chronic mild stress (23). Yu et al. (24) also found that BA prevents neuron apoptosis in rats subjected to CUMS. However, the mechanism(s) for these effects is not very clear.

We hypothesized that BA influences oxidative stress and inflammatory cytokines in rodents subjected to CUMS by regulating the NMDAR/NR2B-ERK1/2 signaling. To test this, we assayed the activation of this pathway as well as depression-like behaviors and reactive oxygen species (ROS), and cytokine levels in rodents exposed to CUMS.

## Material and Methods

### Reagents

BA, 3-(4,5-dimethylthiazol-2-yl)-2,5-diphenyltetrazolium bromide (MTT), corticosterone, dimethyl sulfoxide, and Fura-2/AM were obtained from Sigma-Aldrich (USA). Fluoxetine (Flu) was purchased from Xiansheng Drug Store (Nanjing, China). Enzyme-linked immunosorbent assay (ELISA) kits for IL-1β (KGEMC001b), IL-6 (KGEMC004), and TNF-α (KGEMC102a) were obtained from Nanjing KeyGen Biotech, Co., Ltd. (China). Fetal bovine serum and high-glucose Dulbecco's modified Eagle's medium were from Gibco-BRL (USA). The bicinchoninic acid protein assay kit was obtained from Nanjing Jiancheng Institute of Bioengineering (China). Primary antibodies, including NR2B (#4207), Ca2^+^/calmodulin-dependent protein kinase II (CaMKII; #4436), phosphorylated ERK1/2 (P-ERK1/2; #4370), ERK (#4695), and glyceraldehyde-3-phosphate dehydrogenase (GAPDH; #2118), were purchased from Cell Signaling Technology (USA).

### Animals

Forty C57BL/6 male mice (7 to 8 weeks old) were purchased from the Zhejiang Animal Center (China). The animals were kept in a conventional animal facility with a 12 h light/12 h dark cycle at a constant temperature of 22-24°C. The mice had free access to water and standard food pellets. All animal experiments were performed according to protocols approved by Tongde Hospital of Zhejiang Province and approved by the Ethics Committee of the Institution for Research and Laboratory Animal Use (No. 201711R035).

### CUMS and experimental design

**Figure 1.**
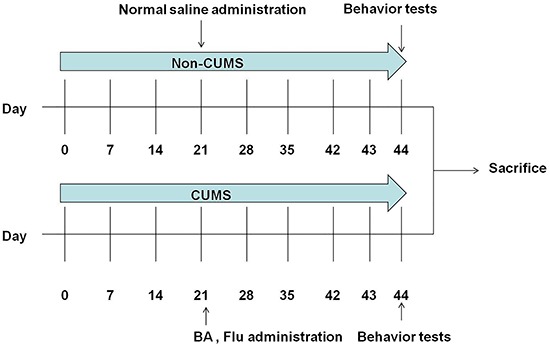
Schematic representation of the experimental procedure for chronic unpredictable mild stress (CUMS) depression model and treatments. BA: baicalin; Flu: fluoxetine.

Mice were randomly assigned to the following five groups (n=8/group): control, model (CUMS), CUMS+Flu (20 mg/kg), CUMS+BA (25 mg/kg), and CUMS+BA (50 mg/kg). The experimental procedure for this study is outlined in Figure 1. The 6-week CUMS procedure was conducted according to the method described previously (25). During the 4th to 6th weeks after CUMS, Flu (20 mg/kg) or BA (25 or 50 mg/kg) was given by intragastric administration once a day. The dosages of BA were determined in preliminary experiments. Equal volumes of normal saline were given to mice in the control and model groups. Behavior tests were performed 24 h after the last CUMS exposure. The animals selected for the hippocampal analyses had statistically significant results from the behavior tests.

### Sucrose preference test

To assess anhedonia, a symptom of depression, the consumption of sucrose was assessed via the sucrose preference test, as described previously (26).

### Open field test

To assess anxiety, mice were placed in the center of an open field apparatus comprising a square wooden arena (40×60×50 cm) with a black surface covering the inside walls. The numbers of crossings into symmetrical sectors by each mouse over 6 min were counted by a trained observer blinded to the experimental group.

### Tail suspension test (TST) and forced swimming test (FST)

The TST and FST were used to assess despair. For the TST, the mice were suspended by their tails (∼50 cm above the floor) in an acoustically and visually isolated environment for 6 min via adhesive tape. The FST was performed as previously described (27). Briefly, each mouse was placed in a glass cylinder (20 cm in height, 14 cm in diameter) filled with water (25±2°C) to a height of 10 cm. Immobility was defined as floating in the water with only small movements necessary to keep their heads above water. For both tests, the duration of immobility was measured during the last 4 min of the 6-min testing period.

### Culture and treatment of PC12 cells

PC12 cells are neuron-like cells that express high levels of the renal glucocorticoid receptors that contribute to nervous system development (28). PC12 cells were cultured in Dulbecco's modified Eagle's medium containing 10% fetal bovine serum and 1% antibiotics (penicillin/streptomycin) in a humidified incubator containing 95% air and 5% CO_2_ at 37°C. To test the effects of BA *in vitro*, PC12 cells were treated first with corticosterone (800 µM) for 6 h and then with various concentrations of BA (20, 40, and 80 µM) for 24 h; the supernatant and cells were collected for the following analyses.

### MTT assay

Cell viability was evaluated in MTT assays. PC12 cells were treated for 2 h with different concentrations of BA (5, 10, 20, 40, 80, and 160 µM) and then incubated with 20 µL of MTT (5 mg/mL) for 4 h. The culture medium was then discarded, and 150 µL of dimethyl sulfoxide was added. The absorbance values at 570 nm (A_570_) were measured for each group using a microplate spectrophotometer. The percent cell viability was calculated as the mean A_570_ (treated) / mean A_570_ (control) × 100. Experiments were conducted in triplicate.

### Measurement of intracellular calcium

Intracellular Ca^2+^ concentrations ([Ca^2+^]_i_) in PC12 cells were assessed by Fura-2/AM fluorescence. Briefly, PC12 cells were collected by centrifugation at 1,200 *g* for 10 min at 4°C and incubated at 37°C for 1 h with Fura-2/AM (5 mM), and then centrifuged twice at 1,200 *g* for 4 min at 4°C. The cells were resuspended in HEPES buffer (137 M NaCl, 5 mM KCl, 1 mM MgCl_2_, 1.5 mM CaCl_2_, 10 mM HEPES, and 25 mM d-glucose, pH 7.4), and fluorescence was measured in a microplate reader using excitation and emission wavelengths of 340 and 500 nm, respectively.

### Measurement of intracellular ROS

Intracellular ROS levels were measured using 2′,7′-dichlorofluorescein diacetate, a non-fluorescent compound that is enzymatically converted to strongly fluorescent 2′,7′-dichlorofluorescein in the presence of ROS. Briefly, PC12 cells were seeded in a 6-well culture plate at a density of 6×10^5^ cells/well. At the end of treatment, the cells were washed with D-Hank's buffer and incubated with 2′,7′-dichlorofluorescein diacetate (10 µM) for 30 min at 37°C in the dark. After washing three times with phosphate-buffered saline, 2′,7′-dichlorofluorescein fluorescence was measured with a microplate reader using excitation and emission wavelengths of 485 and 538 nm, respectively. The intracellular ROS levels are reported as a percentage of the control.

### Analysis of proinflammatory cytokines

The levels of the proinflammatory mediators IL-1β, IL-6, and TNF-α in PC12 supernatants as well as in serum and hippocampal tissues from animals sacrificed under general anesthesia with pentobarbital were assessed by ELISA (Nanjing KeyGen Biotech, Co., Ltd.) according to the manufacturer's instructions. The results are reported as picograms per milliliter or milligram of protein.

### Western blot analysis

PC12 cells and hippocampal tissue samples were homogenized in ice-cold RIPA buffer (with 0.1% phenylmethylsulfonyl fluoride) and then centrifuged at 1,2000 *g* for 15 min at 4°C; protein concentrations were determined using a bicinchoninic acid kit. Proteins were separated by sodium dodecyl sulfate-polyacrylamide gel electrophoresis and transferred to polyvinylidene difluoride membranes. The membranes were then blocked with 5% skim milk and incubated overnight at 4°C with separate primary antibodies (1:1,000). The membranes were then washed in Tris-buffered saline containing Tween 20 and incubated at room temperature with a secondary antibody (1:1,000). The bands were visualized on a ChemiDoc XRS system (Bio-Rad, USA). Densitometric analyses were performed using ImageJ software (National Institutes of Health, USA).

### Statistical analysis

All data are reported as means±SE. Differences between groups were assessed using one-way analysis of variance with Tukey's multiple comparison tests. Analyses were performed using SPSS 17.0 (SPSS Inc., USA). A P value of <0.05 was considered significant.

## Results

### Effects of BA on depression-related behaviors

**Figure 2.**
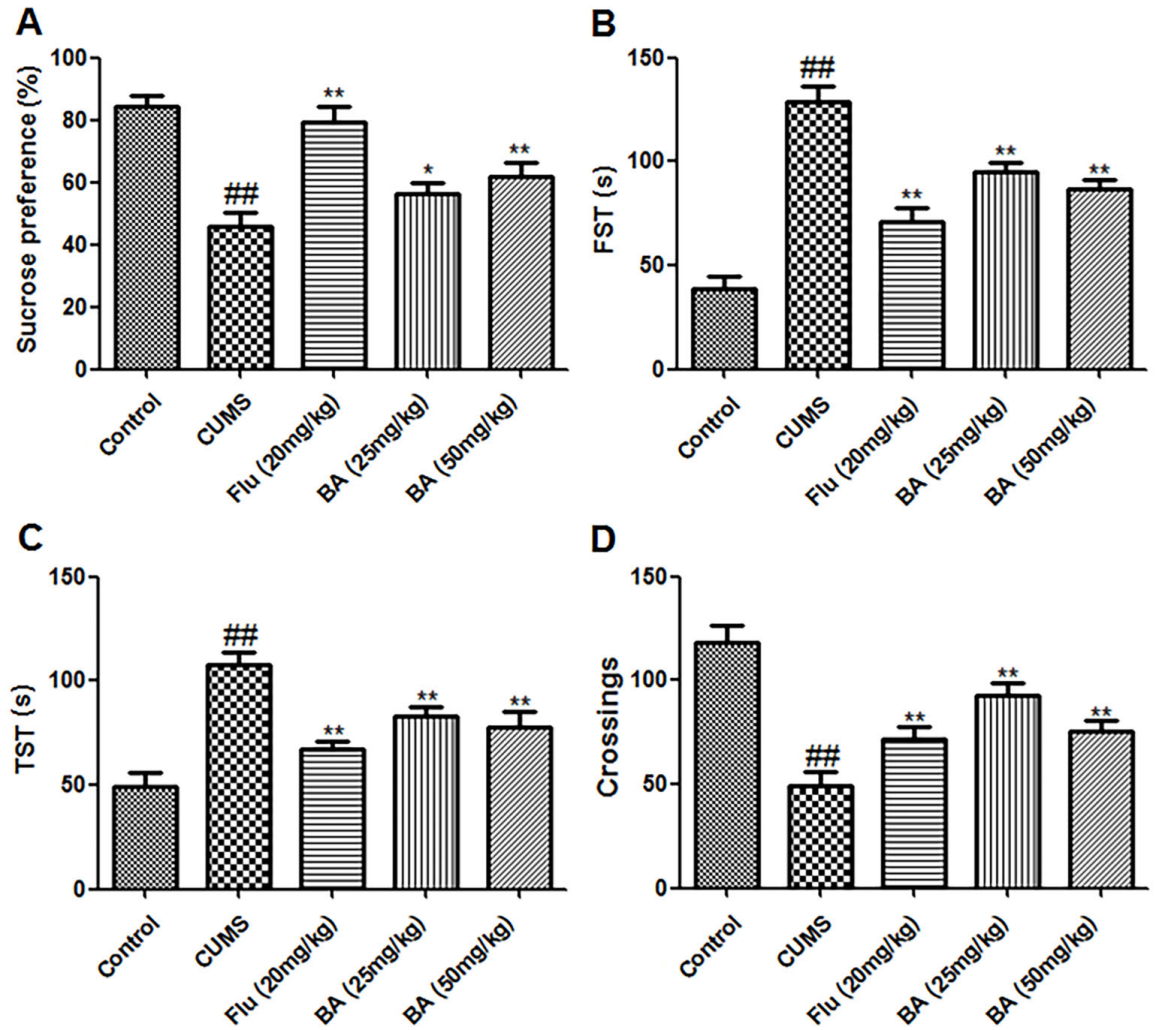
Effects of baicalin (BA) on sucrose consumption (**A**), the forced swimming test (FST) (**B**), the tail suspension test (TST) (**C**), and the open field test (**D**). Data are reported as the means±SE (n=8). ^##^P<0.01 *vs* control; *P<0.05 and **P<0.01 *vs* CUMS (chronic unpredictable mild stress) (ANOVA). Flu: fluoxetine.

As shown in Figure 2A, mice subjected to CUMS consumed less sucrose than those in the control group (P<0.01, n=8), whereas mice treated with BA (25 and 50 mg/kg) and Flu (20 mg/kg) consumed more sucrose than those in the model group (P<0.05, n=8). Similarly, mice subjected to CUMS were immobile for a longer time in the FST and TST than the control mice (P<0.01, n=8), whereas treatment with BA (25 and 50 mg/kg) and Flu (20 mg/kg) decreased the immobility times compared with control levels (P<0.01, n=8) (Figure 2B and C). Lastly, the locomotor activity of mice after CUMS was lower than that of mice in the control group (P<0.01, n=8); this decrease was alleviated by treatment with BA (25 and 50 mg/kg) and Flu (20 mg/kg) (P<0.01, n=8) (Figure 2D).

### Effects of BA on proinflammatory cytokines

**Figure 3.**
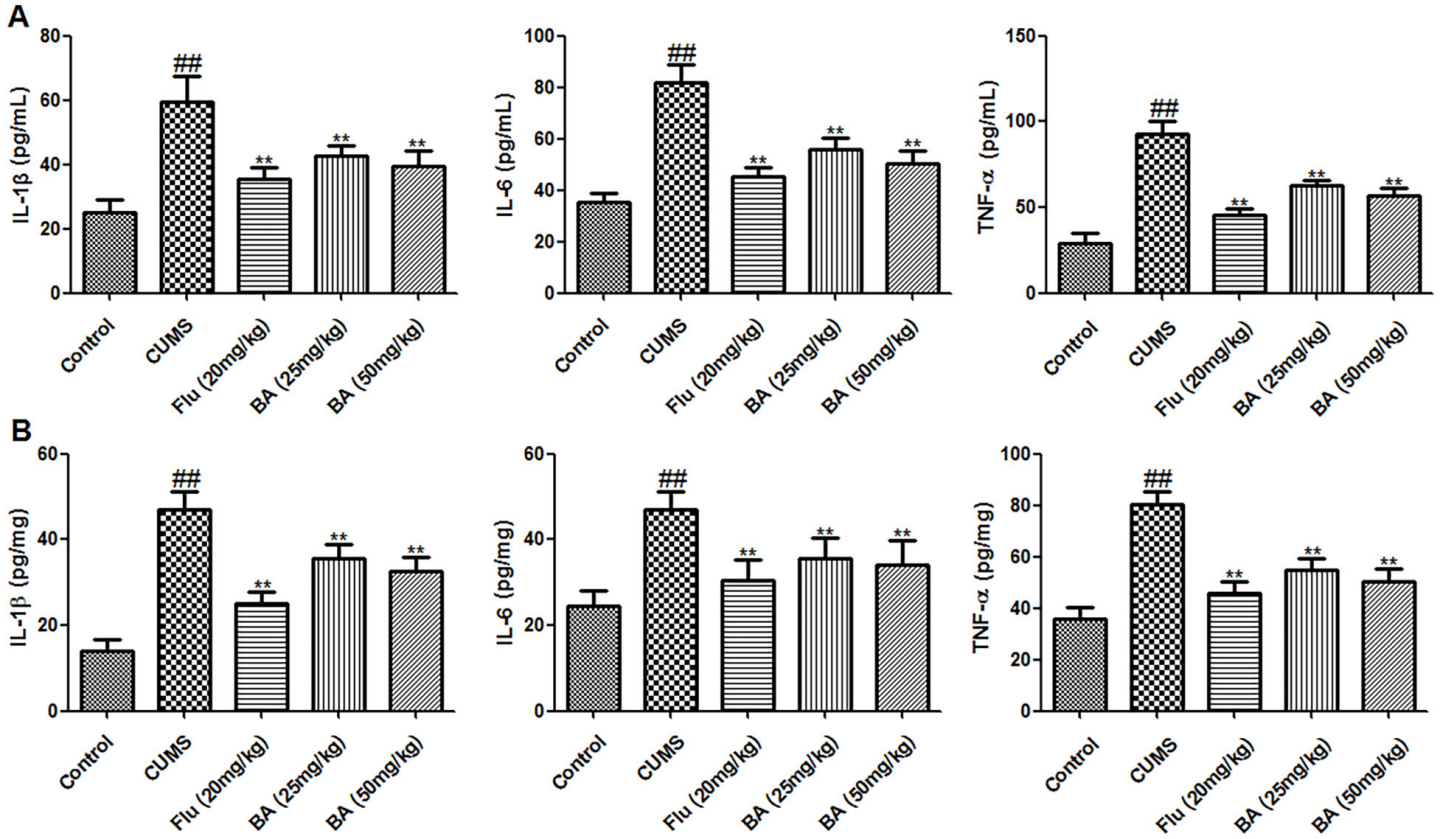
Effects of baicalin (BA) on proinflammatory cytokines in serum (**A**) and the hippocampus (**B**). Data are reported as means±SE (n=8). ^##^P<0.01 *vs* control; **P<0.01 *vs* CUMS (chronic unpredictable mild stress) (ANOVA). Flu: fluoxetine.

ELISAs revealed that CUMS increased the levels of IL-1β, IL-6, and TNF-α both in serum and in the hippocampus (P<0.01, n=8) (Figure 3). These increases were attenuated by treatment with BA (25 and 50 mg/kg) and Flu (20 mg/kg) (P<0.01, n=8).

### Effects of BA on NR2B-ERK signaling

**Figure 4.**
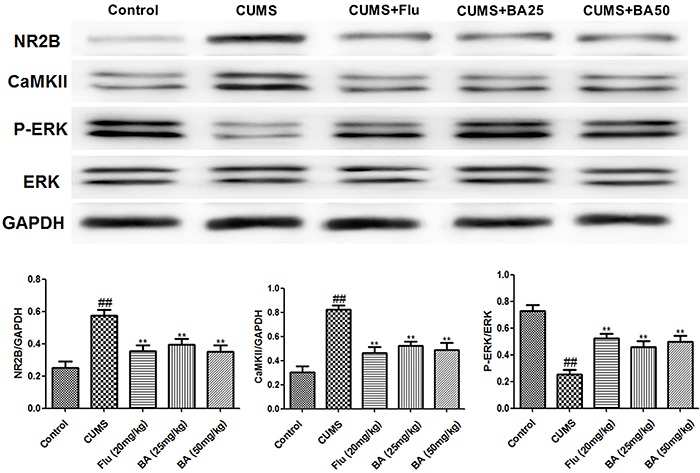
Baicalin (BA) downregulated NR2B and CaMKII and upregulated P-ERK1/2 expression in mice subjected to CUMS (chronic unpredictable mild stress). Data are reported as means±SE (n=8). ^##^P<0.01 *vs* control; **P<0.01 *vs* CUMS (ANOVA). Flu: fluoxetine.

As shown in Figure 4, the levels of NR2B and CaMKII in mice subjected to CUMS were significantly higher than in the control group (P<0.01, n=8); however, the levels were reduced by treatment with BA (P<0.01, n=8). By contrast, the levels of P-ERK1/2 were significantly lower in mice subjected to CUMS than in controls (P<0.01, n=8) but were elevated significantly by BA treatment (P<0.01, n=8).

### Effects of BA on PC12 cells

**Figure 5.**
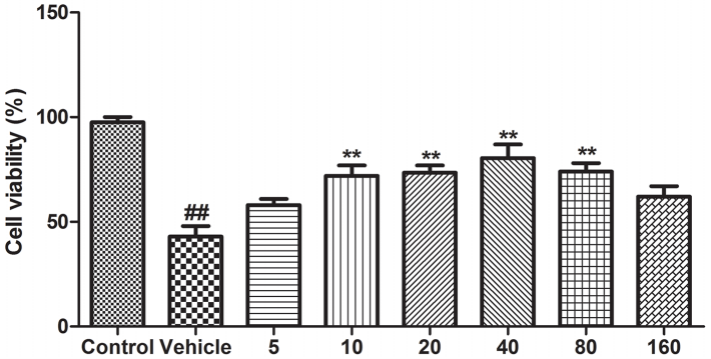
Effects of baicalin (BA) on the viability of PC12 cells. Data are reported as means±SE (n=8). ^##^P<0.01 *vs* control; **P<0.01 *vs* vehicle (ANOVA).

In *in vitro* studies, we found that treatment of PC12 cells with corticosterone reduced their viability significantly (P<0.01, n=8) (Figure 5). However, treatment with BA (10, 20, 40, and 80 µM) attenuated this effect (P<0.01, n=8).

**Figure 6.**
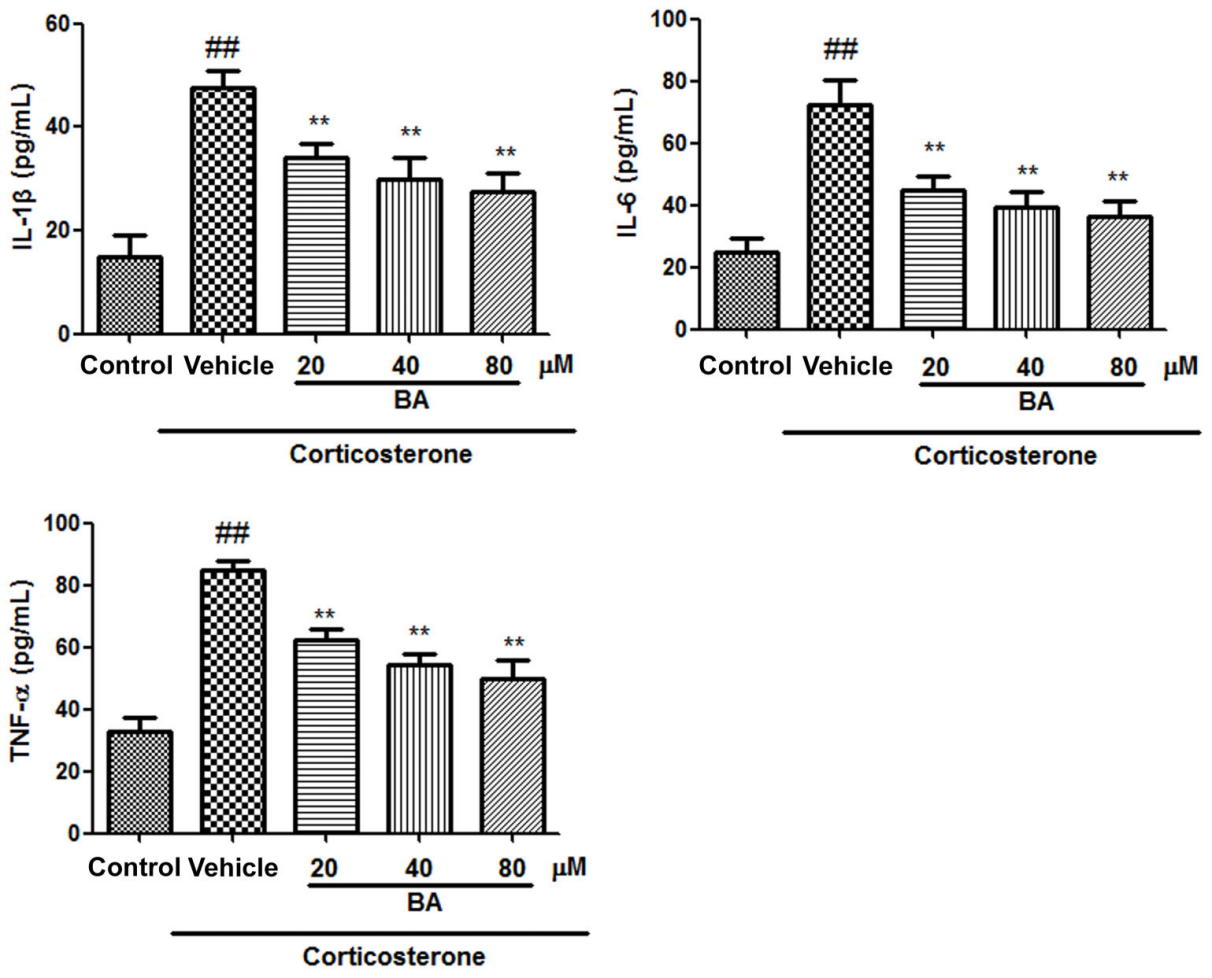
Effects of baicalin (BA) on proinflammatory cytokines in PC12 cells. Data are reported as means±SE (n=8). ^##^P<0.01 *vs* control; **P<0.01 *vs* vehicle (ANOVA).

Consistent with the results from mice subjected to CUMS, the supernatants of PC12 cells treated with corticosterone showed elevated levels of IL-1β, IL-6, and TNF-α (P<0.01, n=8) (Figure 6). These increases were significantly reduced by treatment with BA (20, 40, and 80 µM) (P<0.01, n=8).

**Figure 7.**
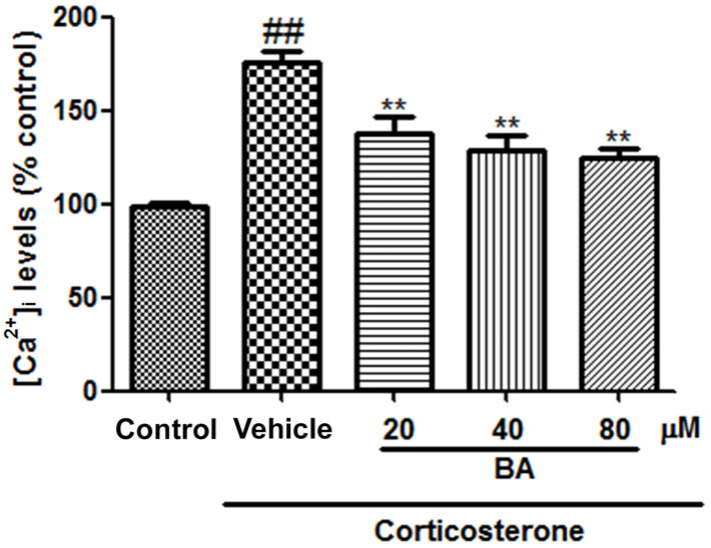
Baicalin (BA) reduced [Ca^2+^]_i_ levels in corticosterone-treated PC12 cells. Data are reported as means±SE (n=8). ^##^P<0.01 *vs* control; **P<0.01 *vs* vehicle (ANOVA).

**Figure 8.**
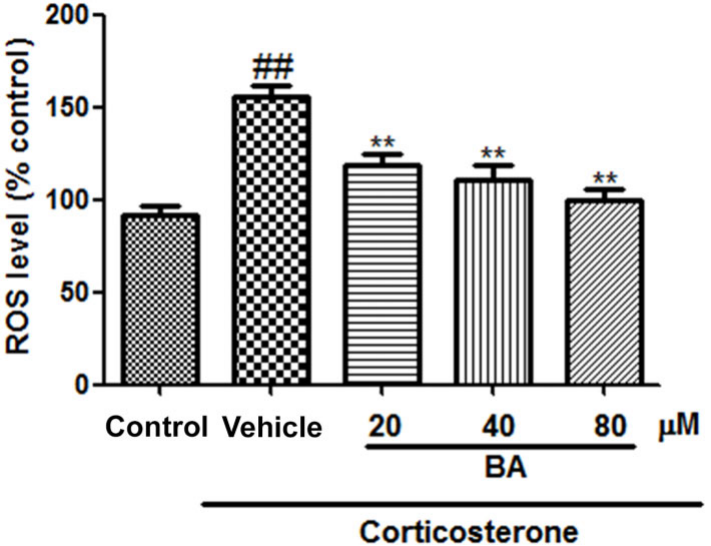
Effects of baicalin (BA) on intracellular reactive oxygen species (ROS) levels in PC12 cells. Data are reported as means±SE (n=8). ^##^P<0.01 *vs* control; **P<0.01 *vs* vehicle (ANOVA).

PC12 cells treated with corticosterone also exhibited increased [Ca^2+^]_i_ and ROS levels (Figures 7 and 8, respectively). These increases were mitigated by treatment with BA (20, 40, and 80 µM) (all P<0.01, n=8).

**Figure 9.**
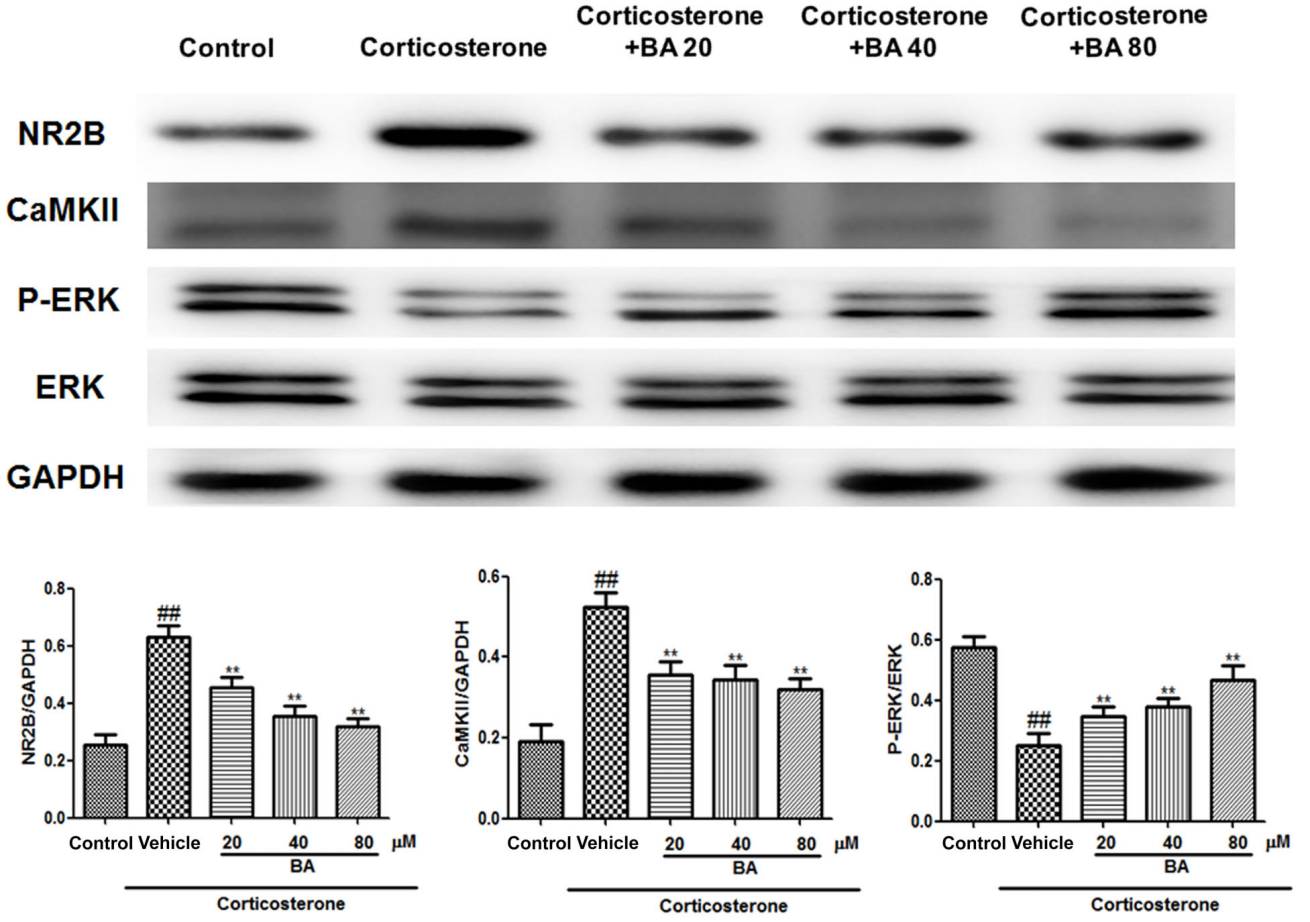
Effects of baicalin (BA) on NR2B, CaMKII, and P-ERK1/2 expression in PC12 cells. Data are reported as means±SE (n=8). ^##^P<0.01 *vs* control; **P<0.01 *vs* vehicle (ANOVA).

Lastly, we observed higher expression of NR2B and CaMKII in PC12 cells treated with corticosterone than in the control group (P<0.01, n=8) (Figure 9). Similarly, these increases were attenuated by BA treatment (P<0.01, n=8). Consistent with the results from mice exposed to CUMS, the levels of P-ERK1/2 were significantly lower in PC12 cells exposed to corticosterone and elevated by BA treatment (P<0.01, n=8).

## Discussion

We investigated the antidepressant-like effects of BA in mice exposed to CUMS and the possible underlying mechanisms. The CUMS model, in which animals are subjected to different stressors on a daily basis (29), is one of the most reliable models of depression and is extensively used to screen antidepressants and investigate the pathophysiology of depression (30). Behavioral experiments are crucial for assessing whether a drug exhibits antidepressant activity, and the results of the present study showed that BA significantly alleviated depression-like behaviors in mice subjected to CUMS. Specifically, BA (similarly to Flu) mitigated the reduced sucrose preference, number of open field crossings in the open field test, and immobility induced by CUMS, thereby demonstrating antidepressant effects.

Increasing evidence indicates that inflammation plays a role in the etiology of major depressive disorder (31). Excessive secretion of proinflammatory cytokines such as IL-1β, IL-6, and TNF-α triggers depression-like behavior; indeed, increased levels of such cytokines are found in the periphery and brains of patients with major depressive disorders (32). Accordingly, mice subjected to CUMS had elevated levels of these cytokines; however, the levels in the BA-treated group were significantly lower than those in the CUMS model group and were similar to the levels in mice given the antidepressant Flu. Similar anti-inflammatory effects of BA were observed in corticosterone-treated PC12 cells, which are neuron-like cells expressing high levels of the renal glucocorticoid receptors that play major roles in nervous system development (33). BA reduced the levels of proinflammatory cytokines and ROS and was neuroprotective against the corticosterone-induced loss of cell viability.

Hippocampal tissues of mice subjected to CUMS, as well as PC12 cells treated with corticosterone, exhibited increased levels of NR2B. NMDAR activation is involved in the neural plasticity and long-term potentiation associated with learning and memory, as well as with certain neurodegenerative diseases (34). These voltage- and ligand-gated channels found throughout the central nervous system regulate intracellular Ca^2+^ transients (35). Activation of NMDARs by glutamate released from presynaptic terminals leads to an influx of calcium. Calcium binds to calmodulin to activate CaMKII and protein kinases A and C, which in turn activate mitogen-activated protein kinases (36) such as ERK1/2, the phosphorylation of which is resistant to protein kinase C inhibition (37). CaMKII, a serine/threonine kinase activated during long-term potentiation via the influx of Ca^2+^ through the NMDARs, is likely of prime importance in terms of linking transient calcium signals to neuronal plasticity (38).

Recently, emerging evidence has revealed that BA promotes neuronal maturation and rescues neurons from apoptosis by inhibiting activation of the GSK3β/NF-κB/NLRP3 signal pathway and prevents the depression-like behaviors in the CUMS animal model (39). BA also modulated APPL2-mediated glucocorticoid receptor hyperactivity and reversed corticosterone-induced depression-like behaviors (40). In the present study, the *in vivo* results indicated that NR2B and CaMKII levels were dramatically increased in the CUMS group compared with those in the control group but were downregulated by BA. In addition, the level of P-ERK1/2 was markedly decreased in the CUMS group. BA treatment attenuated not only the increase in NR2B and CaMKII but also the increase in [Ca^2+^]_i_ and the decrease in ERK1/2 phosphorylation. *In vitro*, BA significantly alleviated corticosterone-induced damage to PC12 cells, as evidenced by the enhanced viability of PC12 cells, reduced IL-1β, TNF-α, and IL-6 levels and expression of NMDAR/NR2B and CaMKII, and increased phosphorylation of ERK1/2. Furthermore, the [Ca^2+^]_i_ and ROS fluorescence intensities in PC12 cells increased significantly in the corticosterone group, while BA reversed this change, mitigating oxidative stress.

In summary, BA exhibited antidepressant-like activity in the CUMS model, which was associated with effects on the NMDAR/NR2B-ERK1/2 and CaMKII signaling pathways. The findings contribute to our understanding of the neurobiology of depression and suggest that BA may serve as an antidepressant and neuroprotective agent that inhibits both oxidative stress and neuroinflammation.
